# Computational analysis of a class of singular nonlinear fractional multi-order heat conduction model of the human head

**DOI:** 10.1038/s41598-024-53822-6

**Published:** 2024-02-12

**Authors:** Mohammad Izadi, Abdon Atangana

**Affiliations:** 1 Department of Applied Mathematics, Faculty of Mathematics and Computer, Shahid Bahonar University of Kerman, Kerman, Iran; 2 Faculty of Natural and Agricultural Sciences, University of the Free State, Bloemfontein, South Africa; 3 Department of Medical Research, China Medical University Hospital, China Medical University, Taichung, Taiwan; 4 IT4Innovations, VSB-Technical University of Ostrava, 17. listopadu 2172/15, 70800 Ostrava-Poruba, Czech Republic

**Keywords:** Collocation points, Convergence analysis, Human head, Heat conduction, Liouville–Caputo fractional derivative, Shifted Vieta–Lucas functions, Singular ODEs, Strongly nonlinearity, Mathematics and computing, Physics

## Abstract

The subject of the article is devoted to the development of a matrix collocation technique based upon the combination of the fractional-order shifted Vieta–Lucas functions (FSVLFs) and the quasilinearization method (QLM) for the numerical evaluation of the fractional multi-order heat conduction model related to the human head with singularity and nonlinearity. The fractional operators are adopted in accordance with the Liouville–Caputo derivative. The quasilinearization method (QLM) is first utilized in order to defeat the inherent nonlinearity of the problem, which is converted to a family of linearized subequations. Afterward, we use the FSVLFs along with a set of collocation nodes as the zeros of these functions to reach a linear algebraic system of equations at each iteration. In the weighted $L_2$ norm, the convergence analysis of the FSVLFs series solution is established. We especially assert that the expansion series form of FSVLFs is convergent in the infinity norm with order ${\mathcal {O}(\frac{1}{K^3})}$, where *K* represents the number of FSVLFs used in approximating the unknown solution. Diverse computational experiments by running the presented combined QLM-FSVLFs are conducted using various fractional orders and nonlinearity parameters. The outcomes indicate that the QLM-FSVLFs produces efficient approximate solutions to the underlying model with high-order accuracy, especially near the singular point. Furthermore, the methodology of residual error functions is employed to measure the accuracy of the proposed hybrid algorithm. Comparisons with existing numerical models show the superiority of QLM-FSVLFs, which also is straightforward in implementation.

## Introduction

The study of the heat conduction associated to the human head subject to various environmental temperatures has been attracted by several research scholars over the past decades. From the theoretical point of views, the temperature distribution of the head based on physically realistic assumptions were investigated in^1,2^. This process was modeled as a two-points boundary value problem (BVP) with nonlinearity and singularity, see also^3^. In a simplified form, the governing equation in 1-D steady state and in the spherically symmetrical form is described as follows

1.1 $$\begin{aligned} \frac{d^2T}{d\tilde{r}^2}+\frac{2}{\tilde{r}}\frac{dT}{d\tilde{r}}+\frac{\delta }{K}e^{-\alpha \,T(\tilde{r})}=0,\quad 0<\tilde{r}<R, \end{aligned}$$

subjected to the boundary conditions given as follows

1.2 $$\begin{aligned} \frac{dT}{d\tilde{r}}\Big |_{\tilde{r}=0}=0,\quad -K\frac{dT}{d\tilde{r}}\Big |_{\tilde{r}=R}=\beta (T-T_a)\Big |_{\tilde{r}=R}. \end{aligned}$$

Here, $T(\tilde{r})$ stands for the absolute temperature depending on $\tilde{r}$ as the radial distance measured from the center to the periphery of the head. Moreover, by $K$ we denote the (average) thermal conductivity, $\beta$ represents the heat exchange coefficient, $\delta$ shows the thermogenesis heat production factor, and $\alpha$ is the metabolic thermogenesis slope parameter. Finally, $T_a$ is denoted the ambient temperature. Next, we introduce the following dimensionless variables given by

$$\begin{aligned} H=T/{T_a},\quad \lambda ={R^2\,\delta }/(K\,T_a),\quad r={\tilde{r}}/{R},\quad m=\alpha \,T_a,\quad Bi=R\beta /K. \end{aligned}$$

By utilizing the former relations in (1.1), we arrive at the integer-order model

1.3 $$\begin{aligned} {\left\{ \begin{array}{ll} \frac{d^2H}{dr^2}+\frac{2}{r}\frac{dH}{dr}+\lambda \, e^{-m\,H(r)}=0,\quad 0<r<1,\\ \frac{dH}{dr}\big |_{r=0}=0,\quad \frac{dH}{dr}\big |_{r=1}=Bi(1-H(r)\big |_{r=1}. \end{array}\right. } \end{aligned}$$

Here, the new parameter *Bi* denotes the Biot number, the parameter $\lambda$ represents the thermogenesis heat production factor, and the metabolic thermogenesis slope parameter denoted by $m$. For more information about the preceding equations (1.1) and (1.3) we refer to^1–3^. Generally, one can not find an analytical or a closed-form solution to (1.3) due to the (exponential) nonlinearity term and two different parameters $m,\lambda$ in (1.3). As a consequence, both semi-analytical and numerical solving approaches become a preferred alternative. Various strategies have been developed in the literature for the BVP (1.3) or the related type equations. In^3^, the varational technique proposed to find solutions of (1.3) accurately. By utilizing the maximum principles, an analytical pointwise lower and upper bounds for the exact solutions derived in^4^. The author of^5^ provided a semi-analytical and non-perturbative solution to (1.3). The stochastic scheme utilizing artificial neural networks investigated in^6^. Some other computational techniques have been developed for (1.3) such as the varational iteration scheme^7^, the sinc-Galerkin procedure^8^, the reproducing kernel method^9^, the modified homotopy analysis approach^10^, and the compact finite difference scheme^11^.

In contrast to the traditional definition of the derivative, various concepts of fractional-order derivatives have been defined in the literature. Indeed, a fractional-order system can provide memory effects and produces solutions, which are much closer to the nature of real-life phenomena, see^12,13^. For more information and applications of various fractional models we refer to^14–20^. In this work, we are interested to interpret the derivatives involved in (1.3) in the sense of Liouville–Caputo derivative. To be more precise, the following class of fractional multi-order BVP with singularity and nonlinearity is considered

1.4 $$\begin{aligned} {\left\{ \begin{array}{ll} \displaystyle {{}^\mathrm{{LC}} \mathcal {D}_r^{\gamma }\,H(r)+\frac{A}{r}\,{}^\mathrm{{LC}} \mathcal {D}^{\sigma }_{r}\,H(r)+\lambda \,e^{-m\,H(r)}} =0,\\ H'(0)=0,\quad H'(1)=Bi(1-H(1)), \end{array}\right. } \quad r\in (0,1). \end{aligned}$$

Here, the derivative operators ${}^\mathrm{{LC}} \mathcal {D}_r^{\gamma },{}^\mathrm{{LC}} \mathcal {D}^{\sigma }_{r}$ are taken in the sense of Liouville–Caputo of orders $\gamma \in (1,2]$ and $\sigma \in (0,1]$ respectively. Moreover, the parameter $A$ is a real constant. Also, two model parameters $m,\lambda$ are previously defined in (1.3). It should be emphasized that in addition to the existing nonlinearlty and singularity, the fractional orders $\gamma ,\sigma$ make the proposed model more complicated than (1.3). These hinder us to find a closed-form solution to this class of equations. Therefore, proposing numerical solutions are preferred to deal with (1.4). To the best in our knowledge, the only method developed is the polynomial least-squares scheme^21^ with $A=2$. On the other hand, the human head model using the He’s fractal derivative was considered in^22^. The resulting fractional model solved by the Taylor expansion series together with two-scale transform technique.

The aim of this research study is to design not only an efficient but also an accurate procedure based on a novel family of polynomial functions to solve (1.4) numerically. Our strategy is relied on a matrix collocation technique utilizing a generalized version or a fractional-order counterpart of the basis functions. The successful applications of the collocation methods with exponential convergence properties and using various bases have been proven in solving many important and practical problems. Among others, we refer to collocation matrix procedures based on Bessel, Chebyshev, Jacobi, and Legendre functions^23–28^, to name a few. The bases will be used here are the Vieta–Lucas polynomials^29^. The shifted form of this set of orthogonal functions has been recently defined in the literature, see cf.^30,31^. However, in the present work we are pioneering in defining and utilizing the fractional-order version of shifted Vieta–Lucas functions (SVLFs) to acquire the approximate solution of (1.4) accurately. On the other hand, we employ the quasilinearization method (QLM) to the model equation (1.4) to have efficiency in our proposed algorithm. The second advantage of the QLM is that we have no longer any nonlinearity term in the model. In other words, we have to solve a set of quasilinear equations in an iterative fashion. Besides the aforementioned benefits of the presented QLM-FSVLFs approach, we also establish the uniformly convergence of the FSVLFs in the $L_{\infty }$ norm. The same convergence property is proved with regard to the $L_2$ norm.

The outline of the current study is considered next. Some concepts of fractional calculus is provided in “Fractional derivative: the Liouville–Caputo operator” section. The definitions of the Vieta–Lucas functions and their shifted and generalized forms are done in “The shifted Vieta–Lucas functions: their generalized form and error estimate” section. The proofs of the convergence of FSVLFs in both weighted $L_2$ and $L_{\infty }$ are then given. In “The combined QLM-FSVLFs procedure” section, we give the details of the main proposed combined QLM-FSVLFs algorithm. The test of accuracy of the presented collocation procedure is accomplished by using the definition of the residual error functions at the end of this section. “Numerical simulations” section is devoted to the computational results in order to verify the efficacy as well as the accuracy of the presented algorithm. Comparison are also made with numerical calculations of available computational algorithms. The last “Conclusions” section contains a conclusion of the study.

## Fractional derivative: the Liouville–Caputo operator

Let us review some fundamental concepts from fractional calculus theory. They are essential in our subsequent discussions in the following sections. For a detailed description we refer the interested readers to the monograph and standard textbook^12,13^.

First, note that by $\Gamma (\cdot )$ we denote the well-known Gamma function. We say that a real-valued function *p*(*r*), $r>0$ belongs to the space $C_{\eta }$, $\eta \in \mathbb {R}$ if there exits a function $k(r)\in {C([0,\infty ))}$ and a real number $\tau >\eta$ such that we have $p(r)=r^{\tau }\,k(r)$. Furthermore, we call $p(r)\in C^m_{\eta }$ if and only if $p^{(m)}(r)\in C_{\eta }$ for a $m\in \mathbb {N}$.

Now, we are in a position to state the definition of the integral operator in the sense of Riemann-Liouville (RL). Let suppose that $p(r)\in C_{\eta }$, $\eta >-1$. The RL integral of function *p*(*r*) of order $\gamma >0$ is defined by

$$\begin{aligned} {}_0 \mathcal {I}_r^{\gamma } p(r)=\frac{1}{\Gamma (\gamma )}\int _0^{r}\frac{p(s)}{(r-s)^{1-\gamma }}ds. \end{aligned}$$

Next, we give the definition of Liouville–Caputo (LC) derivative.

### Definition 2.1

Let $m-1<\gamma <m$, $m\in \mathbb {N}$ and assume that $p(r)\in C_{-1}^{m}$. The LC fractional derivative of *p*(*r*) is defined by

$$\begin{aligned} {}^\mathrm{{LC}} \mathcal {D}^{\gamma }_{r}\,p(r)={}_0 \mathcal {I}_r^{m-\gamma }\,D^{m} p(r)=\frac{1}{\Gamma {(m-\gamma )}}\int _{0}^{r}(r-s)^{m-\gamma -1}p^{(m)}(s)ds,\quad p>0, \end{aligned}$$

where $$D=\frac{d}{dr}.$$

One further remarks that the fractional LC operator ${}^\mathrm{{LC}} \mathcal {D}_r^{\gamma }$ has the linear property. Besides, let $C$ be a real constant number, then one has

2.1 $$\begin{aligned} {}^\mathrm{{LC}} \mathcal {D}^{\gamma }_{r}C=0. \end{aligned}$$

The following property computes the fractional LC derivative of $p(r)=r^{w}$. Thus, we have

2.2 $$\begin{aligned} {}^\mathrm{{LC}} \mathcal {D}_r^{\gamma }\,r^w=\left\{ \begin{array}{ll} 0, &{} \text {if} \hspace{0.5em} w \in \mathbb {N}_{0} \hspace{0.5em} \text {and} \hspace{0.5em}w < \lceil \gamma \rceil ,\\ \displaystyle {\frac{\Gamma {(w +1)}}{\Gamma {(w +1-\gamma )}}r^{w- \gamma }} , &{} \text {if} \hspace{0.5em} w \in \mathbb {N}_{0} \hspace{0.5em}\text {and} \hspace{0.5em}w \ge \lceil \gamma \rceil \hspace{0.5em}\text {or}\hspace{0.5em} w \notin \mathbb {N} \hspace{0.5em}\text {and}\hspace{0.5em} w > \lfloor \gamma \rfloor . \end{array} \right. \end{aligned}$$

Here, $\mathbb {N}_0:=\mathbb {N}\cup \{0\}$. Moreover, $\lceil \xi \rceil$ and $\lfloor \xi \rfloor$ stand for the ceiling and floor functions. While the former outputs the smallest integer number greater or equal than $\xi$, the latter function represents the largest integer number less or equal than $\xi$.

## The shifted Vieta–Lucas functions: their generalized form and error estimate

We begin this section by introducing the definition of shifted Vieta–Lucas functions (SVLFs). These functions were originally introduced in^29^ and their shifted form recently utilized in some papers^30,31^. In the next stage, we define the generalized version of SVLFs. In the last part, we examine the error estimate as well as the convergence properties of SVLFs in a rigorous way.

### The SVLFs and their generalization

The Vieta–Lucas functions on the interval $[-2,2]$ are defined via the following relation

3.1 $$\begin{aligned} \mathcal {V}_k(s)=2\cos \left( k\,\cos ^{-1}\frac{s}{2}\right) , \quad {k\in \mathbb {N}_0}. \end{aligned}$$

Obviously, we have $\mathcal {V}_0(s)=2$. Also, a little manipulation shows that $\mathcal {V}_1(s)=s$. By making use of $s=4r-2$, we get the shifted form of these functions for $r\in [0,1]$. Let us denote the new version by $\mathcal {V}^{\diamond }_k(r)$, which is equal to $\mathcal {V}_k(s)$. By setting $\mathcal {V}^{\diamond }_0(r)=2$ and $\mathcal {V}^{\diamond }_1(r)=-2+4r$, the following recursive formula gives us the remaining SVLFs as

3.2 $$\begin{aligned} \mathcal {V}^{\diamond }_{k+1}(r)=(4r-2)\,\mathcal {V}^{\diamond }_{k}(r)-\mathcal {V}^{\diamond }_{k-1}(r),\quad k=1,2,\ldots . \end{aligned}$$

From (3.2) we get the next two terms of these functions as

$$\begin{aligned} \mathcal {V}^{\diamond }_2(r)=2-16r+16r^2,\quad \mathcal {V}^{\diamond }_3(r)=-2+36r-96r^2+64r^3. \end{aligned}$$

Thus, at $r=0,1$ as the special values we arrive at

$$\begin{aligned} \mathcal {V}^{\diamond }_k(0)=(-2)^k,\quad \mathcal {V}^{\diamond }_k(1)=2. \end{aligned}$$

The proof of the former relations can be easily done by induction on $k$ on the recursive formula (3.2).

Note that the SVLFs solves the following differential equations. In the Sturm-Liouville representation, we can write it as

3.3 $$\begin{aligned} \frac{d}{dr}\Big [(r-r^2)\,w(r)\frac{d}{dr}\mathcal {V}^{\diamond }_k(r)\Big ]=-r^2\,w(r)\,\mathcal {V}^{\diamond }_k(r),\quad k\in \mathbb {N}, \end{aligned}$$

where $w(r):=\left( r-r^2\right) ^{-\frac{1}{2}}$ is known as the weight function. From relation (3.3), one can prove that these functions are orthogonal with regard to *w*(*r*) on $(0, 1)$. The orthogonality condition is

3.4 $$\begin{aligned} \int _{0}^{1} \mathcal {V}^{\diamond }_{k}(r)\,\mathcal {V}^{\diamond }_{k'}(r)\,w(r)\,dr= {\left\{ \begin{array}{ll} 0, &{} k\ne k'\ne 0,\\ 2\pi , &{} k= k'\ne 0,\\ 4\pi , &{} k= k'= 0. \end{array}\right. } \end{aligned}$$

The explicit analytical form of the SVLFs is given by

3.5 $$\begin{aligned} \mathcal {V}^{\diamond }_{k}(r)=2k\,\sum _{\ell =0}^{k}(-1)^{k-\ell }\frac{4^\ell \,(k+\ell -1)!}{(k-\ell )!(2\ell )!}\,r^{\ell },\quad k\in \mathbb {N}. \end{aligned}$$

By setting $\mathcal {V}^{\diamond }_k(r)=0$, we can determine the zeros or roots of SVLFs. The zeros of $\mathcal {V}^{\diamond }_k(r)$ of degree $k\in \mathbb {N}$ are all real and distinct on the open interval $(0, 1)$ given by

3.6 $$\begin{aligned} r_{p}:=\frac{1}{2}+\frac{s^k_p}{4},\quad p=1,2,\ldots ,k, \end{aligned}$$

where $s^k_p:=2\cos \left[ \frac{(2p-1)\,\pi }{2k}\right]$ are the zeros of normal Vieta–Lucas functions $\mathcal {V}_k(s)$ defined in (3.1). Following the previous works^23,32^, the main goal is to employ generalized version of SVLFs to get more accurate results in numerical computations. The next definition will do this task.

#### Definition 3.1

The fractional-order SVLFs (FSVLFs) are denoted by $\mathcal {V}^{\alpha }_k(r)$ on $[0, 1]$ of degree *k*. They are defined through making use of the change of variable $r\rightarrow r^{\alpha }$, $\alpha >0$ as

3.7 $$\begin{aligned} \mathcal {V}^{\alpha }_k(r):=\mathcal {V}^{\diamond }_k(r^{\alpha }),\quad r\in [0,1]. \end{aligned}$$

With the aid of the aforementioned change of variable in (3.5), the following analytical form is obtained

3.8 $$\begin{aligned} \mathcal {V}^{\alpha }_{k}(r)=2k\,\sum _{\ell =0}^{k}(-1)^{k-\ell }\frac{4^\ell \,(k+\ell -1)!}{(k-\ell )!(2\ell )!}\,r^{\ell \alpha },\quad k\in \mathbb {N}. \end{aligned}$$

By a little calculation one can show that the family $\{\mathcal {V}^{\alpha }_k(r)\}_{k=0}^{\infty }$ constitutes a orthogonal set and the related weight function is $w_{\alpha }(r)=\frac{r^{\alpha -1}}{\sqrt{r^{\alpha }-r^{2\alpha }}}$ for $r\in (0,1)$. Thus, we get

3.9 $$\begin{aligned} \int _0^1 \mathcal {V}^{\alpha }_{k}(r)\,\mathcal {V}^{\alpha }_{k'}(r)\,w_{\alpha }(r)\,dr= \frac{\pi }{\alpha } {\left\{ \begin{array}{ll} 0, &{} k\ne k'\ne 0,\\ 2, &{} k= k'\ne 0,\\ 4, &{} k= k'= 0. \end{array}\right. } \end{aligned}$$

The roots of FSVLFs can be utilized as a candidate for the collocation points in our matrix collocation algorithm. Using the fact that the SVLFs of degree *r* can be expressed as its zeros shown in (3.6), we have

$$\begin{aligned} \mathcal {V}^{\diamond }_k(r)=4^k(r-r_1)(r-r_2)\ldots (r-r_k). \end{aligned}$$

Therefore, the roots of $\mathcal {V}^{\alpha }_k(r)$ are defined by $r_p^{1/\alpha }$ for $p=1,2,\ldots ,k$. Thus, we have proved the following fact:

#### Lemma 3.2

The roots of the FSVLFs, $\mathcal {V}^{\alpha }_k(r)$ of degree *k*, are within $(0, 1)$ and given by

3.10 $$\begin{aligned} r_{p}=\left( \frac{1}{2}+\frac{s^k_p}{4}\right) ^{\frac{1}{\alpha }},\quad s^k_p:=2\cos \left[ \frac{(2p-1)\,\pi }{2k}\right] , \end{aligned}$$

for $$p=1,2,\ldots ,k$$.

### Analysis of error and proof of convergence

Next, we are interested to investigate the behavior FSVLFs in an rigorous way. To begin, we recall that a square integrable function *g*(*r*) may be written as a linear combination of FSVLFs. This implies that

3.11 $$\begin{aligned} g(r)=\sum _{k=0}^{\infty } \beta _k\,\mathcal {V}^{\alpha }_k(r),\quad r\in [0,1]. \end{aligned}$$

To acquire the unknown coefficients $\beta _k$, we can employ the orthogonality relation (3.9) to reach

3.12 $$\begin{aligned} \beta _k:=\frac{\alpha }{\pi \,\eta _k}\int _0^1 \mathcal {V}^{\alpha }_{k}(r)\,g(r)\,w_{\alpha }(r)\,dr,\quad k=0,1,2,\ldots , \end{aligned}$$

where $\eta _0=4$ and $\eta _k=2$ for $k>0$. Let us further denote by ${\Vert h \Vert }_{2,w}$ the weighted $L_{2,w}$ norm on $[0, 1]$ with regard to weight function $w_{\alpha }(r)$. To establish that the series solution (3.11) is convergent in the $L_{\infty }$ norm, one requires to prove that the coefficient $\beta _k$ are decreasing functions of $k$. To this end, we first derive an upper bound for $\beta _k$ in (3.11). Let us emphasize that the following results are valid for $\alpha =1$. For a proof related to $\alpha \ne 1$ we refer to^33^.

#### Theorem 3.3

Let a function $g\in L_{2,w}([0,1])\cap C^{(2)}([0,1])$ is represented as (3.11) and $\textrm{M}_{\infty }:=\max _{p\in [0,1]}|g''(p)|$. Then, an upper bound for $\beta _k$ in (3.12) is given as

3.13 $$\begin{aligned} {\vert \beta _k \vert }< \frac{4\textrm{M}_{\infty }}{\pi }\frac{1}{k^{4}},\quad k\ge 2. \end{aligned}$$

#### Proof

By making use of change of variable $r=\frac{1}{2}(1+\cos s)$ or $r=\cos ^{2}\left( \frac{s}{2}\right) =:p(s)$ in (3.12) to render

3.14 $$\begin{aligned} \beta _k=\frac{2}{\pi \eta _k}\int _0^{\pi } \cos (ks)\,g\left( p(s)\right) ds. \end{aligned}$$

Integration by parts (twice) yields

3.15 $$\begin{aligned} \beta _k=\frac{1}{4\pi \,\eta _k}\int _0^{\pi } f_k(s)\,\sin (s)\,g''\left( p(s)\right) \,ds, \end{aligned}$$

where

$$\begin{aligned} f_k(s):=\frac{1}{k}\left( \frac{\sin \left[ (k-1)s\right] }{k-1}-\frac{\sin \left[ (k+1)s\right] }{k+1}\right) . \end{aligned}$$

By using the upper bound for the second derivative we arrive at

3.16 $$\begin{aligned} |\beta _k|\le \frac{\textrm{M}_{\infty }}{4\pi \,\eta _k}\left|\int _0^{\pi } f_k(s)\,ds\right|. \end{aligned}$$

It is now remained to find the value of the integral term in (3.16). By changing of variables $v=\left( k\pm 1\right) s$, we get for an odd $k>1$ as

$$\begin{aligned} \int _0^{\pi } f_k(s)\,ds=\frac{2}{k}\left( \frac{1}{(k-1)^2}-\frac{1}{(k+1)^2}\right) =\frac{8}{(k-1)^2(k+1)^2}. \end{aligned}$$

For $k\ge 2$ we always have $k+1>k-1> k/2$. Consequently, we arrive at

3.17 $$\begin{aligned} \left|\int _0^{\pi } f_k(s)\,ds\right|\le \frac{32}{k^4}. \end{aligned}$$

By inserting (3.17) into (3.16) and using the fact that $\eta _k=2$ for $k\ge 1$, the proof is finished. $\square$

In practice we have to utilize a cutted series representation to approximate *g*(*r*) instead of the infinite series form (3.11). By truncating (3.11) up to its first $(K+1)$ terms, we have

3.18 $$\begin{aligned} g(r)\approx g_K(r)=\sum _{k=0}^{K} \beta _k\,\mathcal {V}^{\alpha }_k(r). \end{aligned}$$

Let assume that two consecutive approximations to *g*(*r*) will be denoted by $g_K(r)$ and $g_{K+1}(r)$. Our goal is to measure the difference between them as

3.19 $$\begin{aligned} e_K(r):=g_{K+1}(r)-g_K(r). \end{aligned}$$

In the next result, we derive an upper bound for the error $e_K$ in the weighted $L_2$ norm.

#### Theorem 3.4

Let suppose the assumptions of Theorem 3.3 are valid. Then, we get an error estimate as

$$\begin{aligned} {\Vert e_K(r) \Vert }_{2,w}< \frac{2\sqrt{2}\textrm{M}_{\infty }}{\sqrt{\pi }}\frac{1}{K^{4}}. \end{aligned}$$

#### Proof

We first exploit the definition of error (3.19) followed by using (3.18) to get

$$\begin{aligned} {\Vert e_K(r) \Vert }_{2,w}&={\Vert g_{K+1}(r)-g_{K}(r) \Vert }_{2,w}\\&=\Big \Vert \sum _{k=0}^{K+1} \beta _k\,\mathcal {V}^{\alpha }_k(r)-\sum _{r=0}^{K} \beta _k\,\mathcal {V}^{\alpha }_k(r)\Big \Vert _{2,w}\\&={\Vert \beta _{K+1}\,\mathcal {V}^{\alpha }_{K+1}(r) \Vert }_{2,w}\\&={\vert \beta _{K+1} \vert }\,{\Vert \mathcal {V}^{\alpha }_{K+1}(r) \Vert }_{2,w} \end{aligned}$$

Applying the orthogonality condition (3.9) reveals that ${\Vert \mathcal {V}^{\alpha }_{K+1}(r) \Vert }_{2,w}=\sqrt{\pi /(2\alpha )}$ for $K\ge 1$. Hence, the result of Theorem 3.3 with $\alpha =1$ gives us

$$\begin{aligned} {\Vert e_K(r) \Vert }_{2,w}&={\vert \beta _{K+1} \vert }\,\sqrt{\frac{\pi }{2\alpha }}\\&<\frac{4\textrm{M}_{\infty }}{\pi } (K+1)^{-4}\,\sqrt{\frac{\pi }{2}}\\&<2\sqrt{2}\textrm{M}_{\infty }\,K^{-4}/(\sqrt{\pi }). \end{aligned}$$

$$\square$$

The error between the cutted (finite) series form $g_{K}(r)$ in (3.18) and the infinite expansion series (3.11) will be defined next by

3.20 $$\begin{aligned} E_K(r)=g(r)-g_{K}(r). \end{aligned}$$

We refer to this error as the global error. We are interested to estimate an upper bound for $E_K(r)$ in both $L_{2,w}$ and $L_{\infty }$ norms. The first upper bound is obtained in the weighted $L_{2,w}([0,1])$ norm.

#### Theorem 3.5

Under the assumptions of Theorem 3.3, the (global) error term $E_K(r)$ in the $L_{2,w}([0,1])$ norm is bounded as

$$\begin{aligned} {\Vert E_{K}(r) \Vert }_{2,w}< \sqrt{\frac{8}{7\pi }}\frac{\textrm{M}_{\infty }}{\sqrt{K^7}}. \end{aligned}$$

#### Proof

In accordance to the definitions (3.11) and (3.18) we get

$$\begin{aligned} {\Vert E_{K} \Vert }_{2,w}^2=\Big \Vert \sum _{k=0}^{\infty }\beta _k\,\mathcal {V}^{\alpha }_k(r)-\sum _{k=0}^{K} \beta _k\,\mathcal {V}^{\alpha }_k(r)\Big \Vert _{2,w}^2=\Big \Vert \sum _{k=K+1}^{\infty }\beta _k\,\mathcal {V}^{\alpha }_k(r)\Big \Vert _{2,w}^2. \end{aligned}$$

The orthogonality condition (3.9) yields

$$\begin{aligned} {\Vert E_{K} \Vert }_{2,w}^2=\frac{\pi }{2\alpha }\sum _{k=K+1}^{\infty } \beta _k^2. \end{aligned}$$

The inequality (3.13) given in Theorem 3.5 is now applied to the foregoing formula. This implies by using $\alpha =1$ that

3.21 $$\begin{aligned} {\Vert E_{K} \Vert }_{2,w}^2\le \frac{\pi }{2}\left( \frac{4\textrm{M}_{\infty }}{\pi }\right) ^2\sum _{k=K+1}^{\infty } \frac{1}{k^{8}}. \end{aligned}$$

If one uses the Integral Test^34^, the immediate conclusion is

$$\begin{aligned} \sum _{k=K+1}^{\infty } \frac{1}{r^{8}}\le \int _K^{\infty } \frac{dt}{t^{8}}=\frac{1}{7K^7}. \end{aligned}$$

We then insert the last inequality into (3.21). Ultimately, we take the square root to get the desire assertion. $\square$

#### Remark 3.6

It should be emphasized that our error bound is of order $\mathcal {O}(K^{-7/2})$. This upper bound is an improvement over the existing upper bounds in the literature^31,35^. In fact, they obtained the error bound as ${\Vert E_{K} \Vert }_{2,w}=\mathcal {O}(K^{-3/2})$ when $\alpha =1$. In particular, we will prove that the norm of the error ${\Vert E_{K} \Vert }_{\infty }=\mathcal {O}(K^{-3})$. This shows that the expansion series solution $g_K(r)$ converges uniformly as $K\rightarrow \infty$.

Still we are interested to estimate the global error (3.20) in the $L_{\infty }$ norm.

#### Theorem 3.7

Suppose that the assumptions of Theorem 3.3 satisfied. Then, the (global) error $E_K(r)=\sum _{k=K+1}^{\infty }\beta _k\,\mathcal {V}^{\alpha }_k(r)$ in the $L_{\infty }([0,1])$ norm has the following bound

3.22 $$\begin{aligned} {\Vert E_{K}(r) \Vert }_{\infty }< \frac{8\textrm{M}_{\infty }}{3\pi }\frac{1}{K^{3}}. \end{aligned}$$

#### Proof

By employing the triangle inequality we get

$$\begin{aligned} {\vert E_K(r) \vert }&\le \sum _{k=K+1}^{\infty }{\vert \beta _k \vert }\,{\vert \mathcal {V}^{\alpha }_k(r) \vert }. \end{aligned}$$

We now use the fact that the Vieta–Lucas functions can be expressible in terms of Cheyshev function of the first kind^29^

$$\begin{aligned} \mathcal {V}_k(s)=2T_k(\frac{s}{2}). \end{aligned}$$

Also, we have that^36^ ${\vert T_r(s) \vert }\le 1$ for all $s\in [-1,1]$ and for all *r*. Therefore, by changing the variable $4r^{\alpha }-2=2\cos s$ we find that ${\vert \mathcal {V}^{\alpha }_k(r) \vert }\le 2$ for all $r\in [0,1]$. Using the upper bound derived in (3.13) we get

3.23 $$\begin{aligned} {\vert E_K(r) \vert }&< 2\left( \frac{4\textrm{M}_{\infty }}{\pi }\right) \sum _{k=K+1}^{\infty }\frac{1}{k^4}. \end{aligned}$$

To estimate $\sum _{k=K+1}^{\infty }\frac{1}{k^4}$, we apply the Integral Test^34^. It follows that

$$\begin{aligned} \sum _{k=K+1}^{\infty } \frac{1}{k^4}\le \int _K^{\infty } \frac{dt}{t^4}=\frac{1}{3K^3}. \end{aligned}$$

Now, the last identity is placed into the inequality (3.23). If we take the supremum over all $r\in [0,1]$, we get the desired assertion. $\square$

## The combined QLM-FSVLFs procedure

We note that the technique of matrix collocation with the aid of FSVLFs can be utilized to solve the nonlinear human head model (1.4) directly. However, the main deficiency of applying the direct method is that the resultant system of equation has nonlinearity. This implies that the spent time to solve the nonlinear system increases as a function of *K*, the number of FSVLFs bases, in order to attain an acceptable degree of accuracy. To overcome the inherit nonlinearity, we propose to apply the technique of quasilinearization to the original model (1.4). It transforms the model under consideration into a sequence of linearized subequations. In accordance to theory of the Newton method by using a rough first approximation, we get a quadratic convergence to the solution of the heat conduction model related human head in model problem (1.4).

Let us describe the basic facts related to the quasilinearization method (QLM). After converting the nonlinear model of human head (1.4) into a set of quasilinear submodels, the direct FSVLFs matrix collocation strategy is applied to every linearized models. In what follows, we refer to this hybrid technique as the QLM-FSVLFs approach. For detailed descriptions and also the recently published papers on the applications of QLM, see cf.^37–40^.

To begin with, we rewrite the nonlinear human head model (1.4) as

4.1 $$\begin{aligned} \mathcal {L}^{\gamma ,\sigma }[H](r)=G(r,H(r)). \end{aligned}$$

Here, $\mathcal {L}^{\gamma ,\lambda }$ denotes the linear operator and *G* is a nonlinear function. They defined by

$$\begin{aligned} \mathcal {L}^{\gamma ,\sigma }[H](r):= \displaystyle {{}^\mathrm{{LC}} \mathcal {D}_r^{\gamma }\,H(r)+\frac{A}{r}\,{}^\mathrm{{LC}} \mathcal {D}^{\sigma }_{r}\,H(r)} ,\quad G(r,H(r)):=-\lambda \,e^{-m H(r)}. \end{aligned}$$

To proceed, we suppose that $H_0(r)$ denotes the rough first approximation to *H*(*r*) as the true solution of (4.1). Now, the process of QLM for (4.1) can be given as

$$\begin{aligned} \mathcal {L}^{\gamma ,\sigma }[H_{\ell +1}](r)\approx G(r,H_\ell (r))+G_{H}(r,H_\ell (r))\Big (H_{\ell +1}(r)-H_\ell (r)\Big ),\quad \ell =0,1,\ldots . \end{aligned}$$

Along with the preceding equations, the same initial conditions as in (1.4) are given by

4.2 $$\begin{aligned} H'_{\ell +1}(0)=0,\quad H'_{\ell +1}(1)=Bi(1-H_{\ell +1}(1)). \end{aligned}$$

Doing some straightforward computations, the application of QLM yields the next expression for (4.1)

4.3 $$\begin{aligned} \mathcal {L}^{\gamma ,\sigma }[H_{\ell +1}](r)-\lambda \,m\,\,e^{-m H_\ell (r)}\,H_{\ell +1}(r)=-\lambda \,e^{-m H_\ell (r)}\Big (1+mH_\ell (r)\Big ), \quad \ell =0,1,\ldots . \end{aligned}$$

For the sake of abbreviation, we make use of the following notations

$$\begin{aligned} \xi _{2,\ell }(r):=\frac{A}{r},\quad \xi _{1,\ell }(r):=-\lambda \,m\,\,e^{-m H_\ell (r)},\quad \theta _\ell (r):=-\lambda \,e^{-m H_\ell (r)}\Big (1+mH_\ell (r)\Big ), \end{aligned}$$

for $\ell =0,1,\ldots$ to write the last equation (4.3) in a compact expression as

4.4 $$\begin{aligned} {}^\mathrm{{LC}} \mathcal {D}_r^{\gamma }\,H_{\ell +1}(r)+\xi _{2,\ell }(r)\,{}^\mathrm{{LC}} \mathcal {D}^{\sigma }_{r}\,H_{\ell +1}(r)+\xi _{1,\ell }(r)\,H_{\ell +1}(r)=\theta _\ell (r),\quad \ell =0,1,\ldots . \end{aligned}$$

The solution of the quasilinear multi-order BVPs (4.4) can now be determined with the help of the FSVLFs matrix collocation strategy. Using the same expansion series (3.18), the approximate solution of (4.4) is taken as a cutted series with $(K+1)$ bases as

4.5 $$\begin{aligned} H_{\ell +1}(r)\approx {h}^{(\ell +1)}_{K,\alpha }(r)=\sum _{k=0}^{K} \beta ^{(\ell )}_k\,\mathcal {V}^{\alpha }_k(r), \end{aligned}$$

for $\ell =0,1,\ldots$. The unknown coefficients $\beta ^{(\ell )}_k$ will put in a vector form compactly as

$$\begin{aligned} \pmb {\pmb {B}}^{(\ell )}_{K}=\left[ \beta ^{(\ell )}_0\quad \beta ^{(\ell )}_1\quad \ldots \quad \beta ^{(\ell )}_K\right] ^T. \end{aligned}$$

The next aim is to form the vector of FSVLFs as

$$\begin{aligned} \pmb {V}^{\alpha }_{K}=\left[ \mathcal {V}^{\alpha }_0(r)\quad \mathcal {V}^{\alpha }_1(r)\quad \ldots \quad \mathcal {V}^{\alpha }_K(r)\right] ^T. \end{aligned}$$

With these two above vectors we can state the approximate solution ${h}^{(\ell +1)}_{K,\alpha }(r)$ in (4.5) in the form

4.6 $$\begin{aligned} {h}^{(\ell +1)}_{K,\alpha }(r)=\pmb {V}^{\alpha }_{K}(r)\,\pmb {\pmb {B}}^{(\ell )}_{K}. \end{aligned}$$

The next Lemma provides a decomposition for $\pmb {V}^{\alpha }_K$. Its proof is easily obtainable by considering the relation (3.8) in Definition (3.1).

### Lemma 4.1

The vector of FSVLFs is represented by

4.7 $$\begin{aligned} \pmb {V}^{\alpha }_K(r)=\pmb {R}^{\alpha }_K(r)\,\pmb {N}_K, \end{aligned}$$

where the vector of monomials is defined by

$$\begin{aligned} \pmb {R}^{\alpha }_K(r)=\left[ 1\quad r^{\alpha }\quad r^{2\alpha }\quad \ldots \quad r^{K\alpha }\right] , \end{aligned}$$

and $\pmb {N}_K$ as an upper triangular matrix has the following structure

$$\begin{aligned} \pmb {N}_K=\begin{pmatrix} 2 &{} -2 &{} 2 &{} \ldots &{} 2(-1)^{K-1} &{} 2(-1)^{K} \\ 0 &{} 4 &{} -16 &{} \ldots &{} 2(K-1)\displaystyle {\frac{4\,(-1)^{K-2}\,(K-1)!}{(K-2)!\,2!}} &{} 2K\displaystyle {\frac{4\,(-1)^{K-1}\,K!}{(K-1)!\,2!}}\\ 0 &{} 0 &{} 16 &{} \ldots &{} 2(K-1)\displaystyle {\frac{4^2(-1)^{K-3}\,(K)!}{(K-3)!\,4!}} &{} 2K\displaystyle {\frac{4^2(-1)^{K-2}\,(K+1)!}{(K-2)!\,4!}} \\ \vdots &{} \vdots &{} \ddots &{} \ddots &{} \ddots &{} \vdots \\ 0 &{} 0 &{} 0 &{} \ldots &{} 2(K-1)\displaystyle {\frac{4^{K-1}(-1)^{0}\,(2K-3)!}{0!\,(2K-2)!}} &{} 2K\displaystyle {\frac{4^{K-1}(-1)^{1}\,(2K-2)!}{1!\,(2K-2)!}}\\ 0 &{} 0 &{} 0 &{} \ldots &{} 0 &{} 2K\displaystyle {\frac{4^{K}(-1)^{0}\,(2K-1)!}{0!\,(2K)!}} \end{pmatrix}. \end{aligned}$$

The next corollary is obtained by combining two former relations (4.6) and (4.7)

### Corollary 4.2

The approximate solution ${h}^{(\ell +1)}_{K,\alpha }(r)$ in (4.5) is rewritten as

4.8 $$\begin{aligned} {h}^{(\ell +1)}_{K,\alpha }(r)=\pmb {R}^{\alpha }_K(r)\,\pmb {N}_K\,\pmb {\pmb {B}}^{(\ell )}_{K}. \end{aligned}$$

### Remark 4.3

It should be stressed that the matrix representation (4.8) is beneficial when we need to have the high-order derivatives of ${h}^{(\ell +1)}_{K,\alpha }(r)$. To do so, we have to calculate the corresponding high-order derivatives of the vector $\pmb {R}^{\alpha }_K(r)$. Owing to the boundary conditions (4.5), we need to compute only the first-order derivative of the unknown solution. Additionally, the other requirements are the computations of the fractional derivatives of order $\gamma$ and $\sigma$ as they are appeared in (1.4). In this respect, we utilize two properties (2.1) and (2.2) as basic tools. According to the previous works^32^ (Algorithm 3.1) or^23^ (Algorithm 4.1), we are able to calculate the $\gamma$- and $\sigma$-derivatives of $\pmb {R}^{\alpha }_K(r)$ efficiently and straightforwardly. The complexity of the proposed algorithm is bounded by $\mathcal {O}(K+1)$. Henceforth, the vector representation forms of the derivatives are denoted by

$$\begin{aligned} \pmb {R}^{(\psi ,\alpha )}_K(r)&:={}^\mathrm{{LC}} \mathcal {D}^{\psi }_{r}\,\pmb {R}^{\alpha }_K(r),\quad \psi =\gamma ,\sigma ,\\ \pmb {R}^{(1,\alpha )}_K(r)&:=\frac{d}{dr}\pmb {R}^{\alpha }_K(r). \end{aligned}$$

Once we have the derivatives of $\pmb {R}^{\alpha }_K(r)$ at hand, we can easily deduce the followings:

### Corollary 4.4

- The first-order derivative of ${h}^{(\ell +1)}_{K,\alpha }(r)$ in (4.8) is given by
  4.9 $$\begin{aligned} \frac{d}{dr}{h}^{(\ell +1)}_{K,\alpha }(r)=\pmb {R}^{(1,\alpha )}_K(r)\,\pmb {N}_K\,\pmb {\pmb {B}}^{(\ell )}_{K}. \end{aligned}$$
- The fractional-order derivatives of ${}^\mathrm{{LC}} \mathcal {D}^{\psi }_{r}\,{h}^{(\ell +1)}_{K,\alpha }(r)$ in (4.8) for $\psi =\gamma ,\sigma$ are computed as
  4.10 $$\begin{aligned} {}^\mathrm{{LC}} \mathcal {D}^{\psi }_{r}\,{h}^{(\ell +1)}_{K,\alpha }(r)=\pmb {R}^{(\psi ,\alpha )}_K(r)\,\pmb {N}_K\,\pmb {\pmb {B}}^{(\ell )}_{K}. \end{aligned}$$

To continue, we utilize the zeros of FSVLFs as defined in (3.10). Note that we have $(K+1)$ unknown coefficients in (4.5). Thus, the zeros of $\mathcal {V}^{\alpha }_{K+1}(r)$ will be utilized as these are labeled as $r_0,r_1,\ldots ,r_K$. We are now in a position to place these nodes into the vector formats of the unknown solutions and its fractional-order derivatives. So, we introduce the following matrix forms

4.11 $$\begin{aligned} \pmb {R}_{\gamma }:=\begin{pmatrix} \pmb {R}^{\gamma }_K(r_0)\\ \pmb {R}^{\gamma }_K(r_1)\\ \vdots \\ \pmb {R}^{\gamma }_K(r_K) \end{pmatrix},\quad \pmb {R}_{\sigma }:= \begin{pmatrix} \pmb {R}^{\sigma }_K(r_0)\\ \pmb {R}^{\sigma }_K(r_1)\\ \vdots \\ \pmb {R}^{\sigma }_K(r_K) \end{pmatrix},\quad \pmb {R}:=\begin{pmatrix} \pmb {R}_K(r_0)\\ \pmb {R}_K(r_1)\\ \vdots \\ \pmb {R}_K(r_K) \end{pmatrix}. \end{aligned}$$

We thus reach at the next assertion:

### Lemma 4.5

In the vector formats, the approximate solution ${h}^{(\ell +1)}_{K,\alpha }(r)$ and its fractional $\psi$-derivatives ${}^\mathrm{{LC}} \mathcal {D}^{\psi }_{r}\,{h}^{(\ell +1)}_{K,\alpha }(r)$ for $\psi =\sigma ,\gamma$ computed at the zeros of FSVLFs can be represented as

4.12 $$\begin{aligned} \begin{aligned} \pmb {H}_{\ell +1}&=\pmb {R}\,\,\pmb {N}_K\,\pmb {\pmb {B}}^{(\ell )}_{K},\\ \pmb {H}^{\psi }_{\ell +1}&=\pmb {R}_{\psi }\,\,\pmb {N}_K\,\pmb {\pmb {B}}^{(\ell )}_{K}, \end{aligned} \end{aligned}$$

where the vectors of $\pmb {R}$ and $\pmb {R}_{\psi }$ have already defined by (4.11) and

4.13 $$\begin{aligned} \pmb {H}_{\ell +1}:=\begin{pmatrix} {h}^{(\ell +1)}_{K,\alpha }(r_0)\\ {h}^{(\ell +1)}_{K,\alpha }(r_1)\\ \vdots \\ {h}^{(\ell +1)}_{K,\alpha }(r_K) \end{pmatrix},\quad \pmb {H}^{\psi }_{\ell +1}:= \begin{pmatrix} {}^\mathrm{{LC}} \mathcal {D}^{\psi }_{r}\,{h}^{(\ell +1)}_{K,\alpha }(r_0)\\ {}^\mathrm{{LC}} \mathcal {D}^{\psi }_{r}\,{h}^{(\ell +1)}_{K,\alpha }(r_1)\\ \vdots \\ {}^\mathrm{{LC}} \mathcal {D}^{\psi }_{r}\,{h}^{(\ell +1)}_{K,\alpha }(r_K) \end{pmatrix}. \end{aligned}$$

Let us come back to the quasilinear form (4.4). We then insert the roots of FSVLFs into (4.4). By employing relations (4.13), one arrives at the next matrix form

4.14 $$\begin{aligned} \pmb {H}_{\ell +1}^{\gamma }+\pmb {\Xi }_{2,\ell }\,\pmb {H}_{\ell +1}^{\sigma }+\pmb {\Xi }_{1,\ell }\,\pmb {H}_{\ell +1}=\pmb {\Theta }_\ell , \end{aligned}$$

where we have used the following notations

$$\begin{aligned} \pmb {\Theta }_\ell := \begin{pmatrix} \theta _\ell (r_0)\\ \theta _\ell (r_1)\\ \vdots \\ \theta _\ell (r_K) \end{pmatrix},\quad \pmb {\Xi }_{s,\ell }:= \begin{pmatrix} \xi _{s,\ell }(r_0) &{} 0 &{}\ldots &{} 0\\ 0 &{} \xi _{s,\ell }(r_1)&{}\ldots &{} 0\\ \vdots &{} \vdots &{}\ddots &{} \vdots \\ 0 &{} 0 &{} \ldots &{} \xi _{s,\ell }(r_K) \end{pmatrix},\quad s=1,2. \end{aligned}$$

We next employ two relations (4.12). At each iteration $\ell$, we get the fundamental matrix equation given by

4.15 $$\begin{aligned} \Big \{\pmb {R}_{\gamma }+\pmb {\Xi }_{2,\ell }\,\pmb {R}_{\sigma }+\pmb {\Xi }_{1,\ell }\,\pmb {R}\Big \}\,\pmb {\pmb {B}}^{(\ell )}_{K}=\pmb {\Theta }_\ell . \end{aligned}$$

In a more convenient form we have

4.16 $$\begin{aligned} \pmb {X}_\ell \,\pmb {\pmb {B}}^{(\ell )}_{K}=\pmb {\Theta }_\ell , \quad \text{ or } \quad [\pmb {X}_\ell ;\pmb {\Theta }_\ell ], \end{aligned}$$

where

$$\begin{aligned} \pmb {X}_\ell :=\pmb {R}_{\gamma }+\pmb {\Xi }_{2,\ell }\,\pmb {R}_{\sigma }+\pmb {\Xi }_{1,\ell }\,\pmb {R}, \end{aligned}$$

for $\ell =0,1,\ldots$. One still requires to incorporate the given boundary conditions (4.2) into the matrix format. Hence, they will be added to the fundamental matrix relation (4.16). Firstly, we consider the boundary condition $h'_{\ell +1}(0)=0$. It is sufficient to employ the relation (4.9). If we let *r* approaching to zero, we have the matrix format

$$\begin{aligned} \widetilde{\pmb {X}}_{0,\ell }\,\pmb {\pmb {B}}^{(\ell )}_{K}=0,\quad \widetilde{\pmb {X}}_{0,\ell }:=\pmb {R}^{(1,\alpha )}_K(0)\,\pmb {N}_K,\quad \text{ or }\quad [\widetilde{\pmb {X}}_{0,\ell };0]. \end{aligned}$$

For the endpoint boundary condition $h'_{\ell +1}(1)+Bi\,h_{\ell +1}(1)=Bi$, we must use both relations (4.8) and (4.9). By approaching $r\rightarrow 1$ we obtain the matrix format

$$\begin{aligned} \widetilde{\pmb {X}}_{1,\ell }\,\pmb {\pmb {B}}^{(\ell )}_{K}=Bi,\quad \widetilde{\pmb {X}}_{1,\ell }:=\left( \pmb {R}^{(1,\alpha )}_K(1)+Bi\,\pmb {R}_K(1)\right) \,\pmb {N}_K,\quad \text{ or }\quad [\widetilde{\pmb {X}}_{1,\ell };Bi]. \end{aligned}$$

By using these two row matrices $[\widetilde{\pmb {X}}_{0,\ell };0]$ and $[\widetilde{\pmb {X}}_{1,\ell };Bi]$ we will substitute two rows of the fundamental matrix equation $[\pmb {X}_\ell ;\pmb {\Theta }_\ell ]$ in (4.16). We denote the resulting modified system as

4.17 $$\begin{aligned} {[}\widetilde{\pmb {X}}_\ell ;\widetilde{\pmb {\Theta }}_\ell ],\quad \ell =0,1,\ldots . \end{aligned}$$

After solving (4.17), we get the unknown coefficients $\beta ^{(\ell )}_{k}$ for $k=0,1,\ldots ,K$ at each iteration $\ell =1,2,\ldots$ in (4.5). We remark that usually taking $r=5$ is enough to reach the desired accuracy when we solve the system (4.17) through utilizing the QLM-FSVLFs technique.

### The methodology of residual error functions (REFs)

Practically, the analytical or exact solutions to (1.4) are not tractable. Particularly, if we dealing with the fractional orders $1<\gamma \le 2$ and $0<\sigma \le 1$. Therefore, the approach of REFs is employed to calculate the accuracy of the developed QLM-FSVLFs. For this purpose, we insert the obtained approximate solution (4.5) into (1.4). Thus, the REFs given by

4.18 $$\begin{aligned} {\mathbb {R}}_{K,\alpha }^{(\ell +1)}(r):=\Big | \displaystyle {{}^\mathrm{{LC}} \mathcal {D}_r^{\gamma }\,{h}^{(\ell +1)}_{K,\alpha }(r)+\frac{A}{r}\,{}^\mathrm{{LC}} \mathcal {D}^{\sigma }_{r}\,{h}^{(\ell +1)}_{K,\alpha }(r)+\lambda \,e^{-m{h}^{(\ell +1)}_{K,\alpha }(r)}} \Big |\cong 0, \end{aligned}$$

for $\ell =0,1,2,\ldots$. Next, the maximum values the REFs (for a fixed $\ell$) is calculated by defining

4.19 $$\begin{aligned} \mathcal {E}_{\infty }\equiv \mathcal {E}_{\infty }^K:=\max _{r\in [0,1]}{{\mathbb {R}}_{K,\alpha }^{(\ell +1)}(r)}. \end{aligned}$$

Finally, we calculate the notion of numerical order of convergence ($\text{ ord}_K^{\infty }$). It is defined by the ratio

4.20 $$\begin{aligned} \text{ ord}_K^{\infty }:=\frac{\ln \mathcal {E}_{\infty }^K-\ln \mathcal {E}_{\infty }^{2K}}{\ln 2}. \end{aligned}$$

## Numerical simulations

The goal is here to describe the performance of the developed matrix collocation approach based on the FSVLFs to the human head model (1.4) with singularity and multi-order fractional derivatives. For this purpose, several numerical computations based on MATLAB software version 2021a have been provided to show the basic fundamental results obtained in this manuscript. Diverse values of parameters $\lambda ,m$ as well as different fractional orders $\gamma ,\sigma$ are considered to illustrate the utility of the QLM-FSVLFs technique. Below, the parameter $\ell$ in the QLM is set as $\ell =5$ in the practical computations. We further use the initial approximation $H_0(r)$ as the zero function.

### The integer-orders $\gamma =2$ and $\sigma =1$

As a starting point, we first consider the integer-order derivatives. Note that we always use $\alpha =1$ when the derivatives are both integers. To continue, we take the next parameters for the human head model (1.4) as

$$\begin{aligned} A=2,\quad \lambda =1,\quad Bi=1. \end{aligned}$$

By utilizing the QLM-FSVLFs procedure with $K=6$ we obtain

$$\begin{aligned} {h}^{(6)}_{6,1}(r)&=- 0.00002031222803\,r^6 + 0.00000919680423\,r^5 - 0.0008265202274\,r^4\\&\quad +\, 0.000004263676977\,r^3- 0.05220576355\,r^2 + 1.160819842. \end{aligned}$$

**Figure 1:**
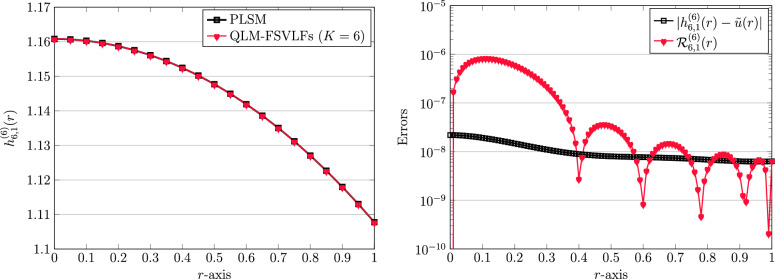
Graphical representation of numerical solutions utilizing QLM-FSVLFs and PLSM (left) and related achieved errors (right) for $\lambda ,m=1$, $K=6$, and with integer-orders $\gamma =2,\sigma =1$.

The above approximate solution is depicted in Fig. 1. To validate our finding, we compare our outcomes with those reported by the polynomial least-squares method (PLSM)^21^ with the same number of basis functions; $K=6$. The reported approximation is as follows

$$\begin{aligned} \tilde{u}(r)=1.16081982 -0.05220539\,r^2 + 0.00000296\,r^3-0.00082464\,r^4 + 0.00000795\,r^5 -0.00002000\,r^6. \end{aligned}$$

A simple comparison indicates that the results of both methods are very close together. This fact can be further justified by computing the difference between two approximate solutions via ${\vert {h}^{(6)}_{6,1}(r)-\tilde{u}(r) \vert }$. This error is shown in Fig. 1, on the right panel. We also visualize the REFs ${\mathbb {R}}_{6,1}^{(6)}(r)$ achieved via (4.18) as depicted in the former figure.

**Figure 2:**
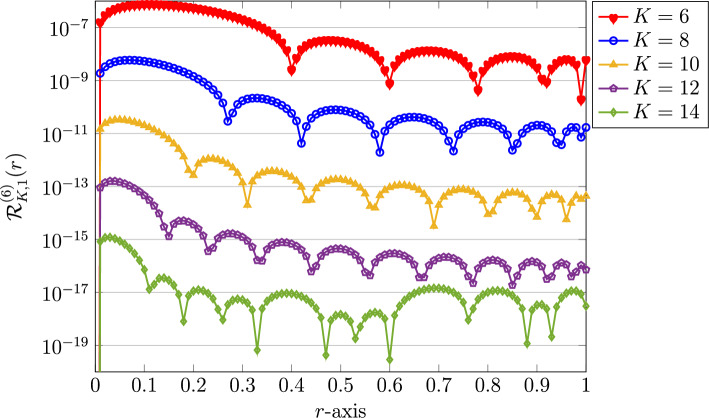
Graphical representation of REFs utilizing QLM-FSVLFs technique for $\lambda ,m=1$, various $K=6,8,10,12,14$, and with integer-orders $\gamma =2,\sigma =1$.

We next utilize larger values of $K=8,10,12,$ and $K=14$ in the computational experiments to justify that the presented QLM-FSVLFs approach converges numerically toward to the actual solution. The achieved REFs related to these values are presented in Fig. 2. It can be readily seen that the REFs is a decreasing function of *K* as increased.

We further validate the computational outcomes gotten by the above aforementioned parameters. To do so, we compare the outcomes with those reported by the well-established existing numerical models as observed in Tables 1 and 2. The first approach is the PLSM^21^ with polynomial of degree six while the second one is the stochastic based on an unsupervised artificial neural networks optimized with genetic algorithms (GAs), active-set technique (AST), and their hybrids named GA-AST from^6^ with ten neurons. Our presented results are obtained by utilizing $K=6,8$, and $K=10$. For justification, the related achieved REFs are also tabulated in these tables. One can clearly infer that our results with $K=6$ are comparable with the outcomes of the PLSM in terms of accuracy. However, our results are more accurate using $K=8$ with those errors reported by the ANN-based procedures in Table 2.

**Table 1:** Comparing of numerical solutions/REFs using QLM-FSVLFs technique for $\gamma =2,\sigma =1$, $\lambda ,m=1$, $K=6,10$, and various $r\in [0,1]$.

*r*	QLM-FSVLFs				PLSM^21^	
	$h^{(6)}_{6,1}(r)$	${\mathbb {R}}_{6,1}^{(6)}(r)$	$h^{(6)}_{10,1}(r)$	${\mathbb {R}}_{10,1}^{(6)}(r)$	Six-degree poly.	Error
0.0	1.16081984	$0.0000_{-0}$	1.160819819590251	$0.0000_{-0}$	1.16081983	$0.000_{-0}$
0.1	1.16029771	$8.4065_{-7}$	1.160297689006561	$2.0152_{-11}$	1.16029769	$6.725_{-8}$
0.2	1.15873032	$6.1609_{-7}$	1.158730315153458	$2.6535_{-13}$	1.15873032	$1.978_{-8}$
0.3	1.15611475	$2.2439_{-7}$	1.156114745851976	$1.3948_{-13}$	1.15611475	$4.388_{-8}$
0.4	1.15244604	$2.7801_{-9}$	1.152446041158113	$2.2824_{-13}$	1.15244604	$5.158_{-9}$
0.5	1.14771725	$3.4620_{-8}$	1.147717243637097	$1.8269_{-13}$	1.14771725	$2.308_{-8}$
0.6	1.14191934	$8.4479_{-10}$	1.141919336227521	$9.1822_{-14}$	1.14191934	$2.141_{-9}$
0.7	1.13504119	$1.4206_{-8}$	1.135041187162281	$1.9870_{-14}$	1.13504119	$4.642_{-8}$
0.8	1.12706948	$4.4394_{-9}$	1.127069481241230	$8.9165_{-15}$	1.12706949	$2.416_{-8}$
0.9	1.11798864	$3.4191_{-9}$	1.117988636563056	$7.0406_{-15}$	1.11798864	$8.311_{-8}$
1.0	1.10778071	$6.4787_{-9}$	1.107780705616379	$4.3711_{-14}$	1.10778071	$4.629_{-9}$

**Table 2:** Comparing of numerical solutions/REFs using QLM-FSVLFs technique for $\gamma =2,\sigma =1$, $\lambda ,m=1$, $K=8$, and various $r\in [0,1]$.

*r*	QLM-FSVLFs		ANN^6^					
	$h^{(6)}_{8,1}(r)$	${\mathbb {R}}_{8,1}^{(6)}(r)$	GA	Error	AST	Error	GA-AST	Error
0.0	1.160819819659	$0.0000_{-0}$	1.16093104	$1.4441_{-4}$	1.16081999	$7.3822_{-8}$	1.16081956	$2.5447_{-7}$
0.1	1.160297689045	$5.3263_{-9}$	1.16040234	$6.4940_{-4}$	1.16029785	$7.6417_{-7}$	1.16029763	$2.3650_{-6}$
0.2	1.158730315169	$1.4361_{-9}$	1.15880759	$4.4537_{-4}$	1.15873046	$8.3864_{-7}$	1.15873036	$2.5593_{-6}$
0.3	1.156114745862	$1.7157_{-10}$	1.15616949	$2.2727_{-4}$	1.15611490	$7.4055_{-7}$	1.15611477	$2.6446_{-7}$
0.4	1.152446041165	$5.7457_{-11}$	1.15249490	$7.0943_{-4}$	1.15244622	$1.0099_{-6}$	1.15244604	$1.5218_{-6}$
0.5	1.147717243642	$7.6349_{-11}$	1.14777601	$6.8283_{-4}$	1.14771742	$1.3692_{-6}$	1.14771726	$4.0837_{-7}$
0.6	1.141919336231	$2.1342_{-11}$	1.14199556	$1.7497_{-4}$	1.14191949	$4.4069_{-7}$	1.14191936	$9.1817_{-7}$
0.7	1.135041187165	$1.9892_{-11}$	1.13513234	$5.3114_{-4}$	1.13504133	$2.0408_{-6}$	1.13504121	$4.1913_{-7}$
0.8	1.127069481243	$2.6313_{-11}$	1.12716571	$9.8891_{-4}$	1.12706964	$5.5233_{-7}$	1.12706950	$6.4930_{-7}$
0.9	1.117988636564	$2.0657_{-11}$	1.11807882	$6.5764_{-4}$	1.11798881	$2.9974_{-6}$	1.11798866	$1.1377_{-7}$
1.0	1.107780705617	$1.6943_{-11}$	1.10786089	$8.4893_{-6}$	1.10778087	$2.5239_{-9}$	1.10778073	$4.4627_{-10}$

The behavior of the obtained errors $\mathcal {E}_{\infty }$ in the QLM-FSVLFs are also examined using the above model parameters as seen in Table 3. The corresponding numerical order of convergence, i.e., $\text{ ord}_R^{\infty }$, are reported alongside in Table 3. Here, we set $K=2,4,8,16$ and the relation (4.20) is utilized. In the case of $m=1$ and for each $K$, the (spent) CPU times measured in seconds are further presented in Table 3. One should note that the needed CPU time is just the time required to solve the modified fundamental system of equations $[\widetilde{\pmb {X}}_\ell ;\widetilde{\pmb {\Theta }}_\ell ]$ in (4.17). The results presented in Table 3 indicate that the developed QLM-FSVLFs with a linear cost produces high-order accurate outcomes.

**Table 3:** The results of $\mathcal {E}_{\infty }$ error norms, the CPU time, and the corresponding $\text{ ord}_R^{\infty }$ for $\lambda =1$, $m=0.5,1,2$, $\gamma =2,\sigma =1$, and different *K*.

*K*	$m=1$			$m=0.5$		$m=2$	
	$\mathcal {E}_{\infty }$	$\text{ ord}_K^{\infty }$	CPU(s)	$\mathcal {E}_{\infty }$	$\text{ ord}_K^{\infty }$	$\mathcal {E}_{\infty }$	$\text{ ord}_K^{\infty }$
2	$6.0552\times 10^{-3}$	−	0.37765	$8.5582\times 10^{-3}$	−	$1.7392\times 10^{-3}$	−
4	$8.8287\times 10^{-5}$	6.0998	0.45875	$1.0514\times 10^{-4}$	6.3469	$1.9290\times 10^{-5}$	6.4945
8	$5.8803\times 10^{-9}$	13.874	0.63598	$4.9241\times 10^{-9}$	14.382	$7.3248\times 10^{-10}$	14.685
16	$1.9231\times 10^{-15}$	21.544	1.06565	$3.2476\times 10^{-15}$	20.532	$7.3347\times 10^{-16}$	19.930

**Figure 3:**
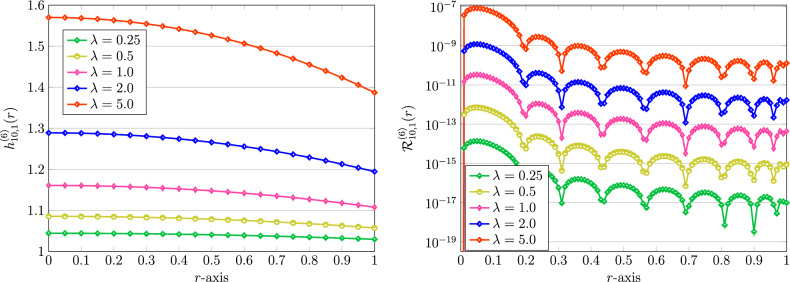
Graphical representation of numerical solution using QLM-FSVLFs procedure (left) and their REFs (right) with fixed $m,Bi=1$, $A=2$, $K=10$, various $\lambda =0.25,0.5,1,2,5$, and with integer-orders $\gamma =2,\sigma =1$.

**Figure 4:**
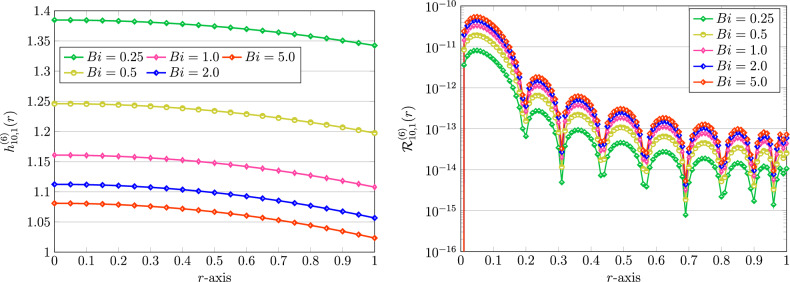
Graphical representation of numerical solution using QLM-FSVLFs procedure(left) and their REFs (right) with fixed $m,\lambda =1$, $A=2$, $K=10$, various $Bi=0.25,0.5,1,2,5$, and with integer-orders $\gamma =2,\sigma =1$.

**Figure 5:**
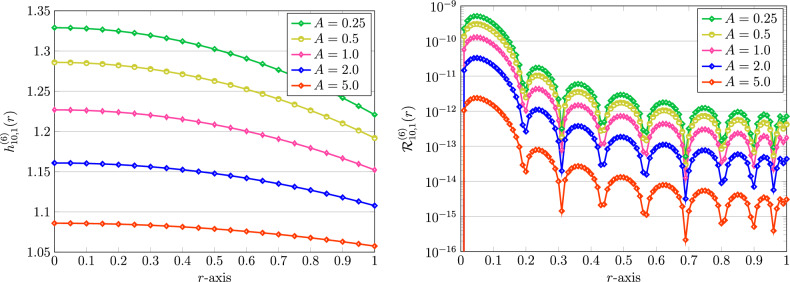
Graphical representation of numerical solution using QLM-FSVLFs technique (left) and their REFs (right) with fixed $m,\lambda ,Bi=1$, $K=10$, various $A=0.25,0.5,1,2,5$, and with integer-orders $\gamma =2,\sigma =1$.

Beside the results shown in Table 3, we next investigate the impact of employing different model parameters $\lambda$, $A$, and *Bi* while the others are assumed to be fixed. To do so, we first fix $m=1$, $A=2$, and $Bi=1$. Figure 3 presents the numerical solutions obtained by various $\lambda =0.25,0.5,1,2$, and $\lambda =5$. The number of bases used here is $K=10$. The achieved REFs associated to these approximate solutions are further visualized in Fig. 3. It can be clearly seen that the REFs are increasing as $\lambda$ is increasing. Next, we fix $m,\lambda =1$, $A=2$ and vary $Bi=0.25,0.5,1,2,5$. The results are shown in Fig. 4. Here, it can be also seen that the REFs are increasing if we increasing the value of the Biot number *Bi* from 0.25 to 5. In the last experiment, we visualize the numerical solutions for different values of the coefficient parameter $A=0.25,0.5,1,2,5$. In these cases, the other parameters are assumed to be fixed as $m,\lambda ,Bi=1$. Figure 5 shows the aforementioned visualizations. Contrary to the other parameters, the REFs will be decreased as *A* increases.

Lastly, for the worst case scenarios, i.e. $\lambda =5$, $Bi=5$, and $A=0.25$ we compute the maximum absolute errors $\mathcal {E}_{\infty }$ as given by (4.19) and the associated numerical order of convergence $\text{ ord}_K^{\infty }$ defined by (4.20). These outcomes are reported in Table 4. One can easily conclude the high-order accuracy of the developed QLM-FSVLFs technique.

**Table 4:** The results of $\mathcal {E}_{\infty }$ error norms and the corresponding $\text{ ord}_R^{\infty }$ for $m,\lambda ,Bi=1$, $A=2$, $\gamma =2,\sigma =1$, and different *K*.

*K*	$\lambda =5$		$Bi=5$		$A=0.25$	
	$\mathcal {E}_{\infty }$	$\text{ ord}_K^{\infty }$	$\mathcal {E}_{\infty }$	$\text{ ord}_K^{\infty }$	$\mathcal {E}_{\infty }$	$\text{ ord}_K^{\infty }$
2	$7.8704\times 10^{-2}$	−	$7.1703\times 10^{-3}$	−	$1.1076\times 10^{-2}$	−
4	$4.0836\times 10^{-3}$	4.2685	$1.1312\times 10^{-4}$	5.9861	$2.8244\times 10^{-4}$	5.2933
8	$3.8235\times 10^{-6}$	10.061	$8.9189\times 10^{-9}$	13.631	$5.4736\times 10^{-8}$	12.333
16	$3.9429\times 10^{-13}$	23.209	$2.0829\times 10^{-15}$	22.030	$1.9878\times 10^{-16}$	28.037

### The fractional-order derivatives

Here and in the following part, let us consider the fractional-order derivatives $\gamma$ and $\sigma$ in the human head model (1.4). Furthermore, the effect of simultaneous use of different parameters $m,\lambda ,Bi,A$, and $\gamma ,\sigma$ on the calculated approximate solutions will be investigated. In particular, the influence of the local parameter $\alpha$ is of important for us. We will present these impacts through figures and tables. For the fractional multi-order model problem (1.4), let us first consider to the next parameters as they recently utilized by^21^

$$\begin{aligned} \gamma =1.7,\quad \sigma =0.7,\quad m=1, \quad \lambda =1,\quad Bi=1,\quad A=2. \end{aligned}$$

Firstly, let us consider $K=6$ and $\alpha =1$. By utilizing the QLM-FSVLFs the approximate solution using these parameters is given by

$$\begin{aligned} {h}^{(6)}_{6,1}(r)&=- 0.01571036495\,r^6 + 0.06711544313\,r^5 - 0.1201035014\,r^4 + 0.1181284766\,r^3\\&\quad -\, 0.1168622159\,r^2 + 1.185870144. \end{aligned}$$

Similar to the integer-order cases, let us mention the numerical results reported by the polynomial least-squares method (PLSM)^21^ with the same number of polynomial basis functions. This approximation was given as

$$\begin{aligned} \tilde{u}(r)=1.18592781- 0.12362096\,r^2 + 0.14497311\,r^3-0.16311024\,r^4 + 0.09852304\,r^5 -0.02436348\,r^6. \end{aligned}$$

**Figure 6:**
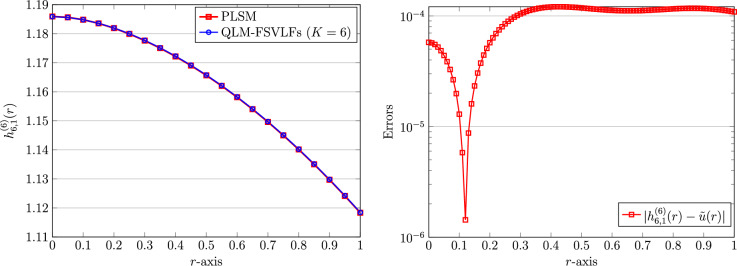
Graphical representation of numerical solutions using QLM-FSVLFs and PLSM (left) and the error between them (right) with $K=6$, $m,\lambda ,Bi=1$, $A=2$, and with fractional-orders $\gamma =1.7,\sigma =0.7$ and $\alpha =1$.

These two approximate solutions are plotted in Fig. 6, on the left picture. To be more precise, we also show the graphical representation of the error between our numerical solution and that solution obtained by PLSM. This error is depicted in Fig. 6, at the right plot.

**Figure 7:**
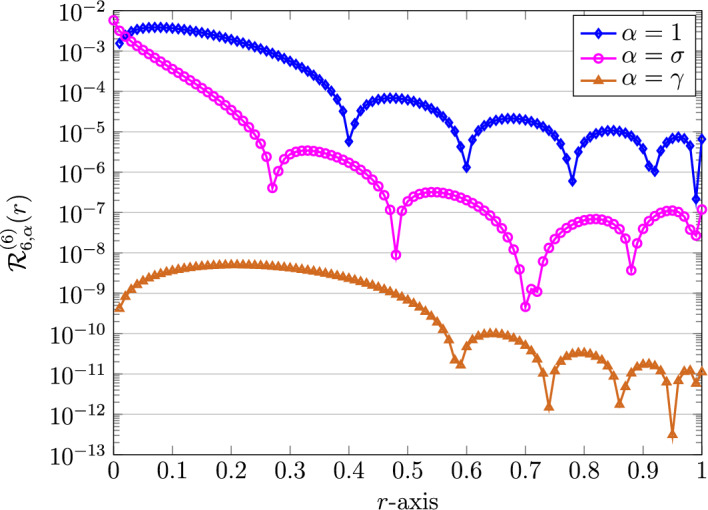
Graphical representation of REFs obtained via QLM-FSVLFs approach for $\lambda ,m,Bi=1$, $A=2$, $K=6$, $\alpha =1,\sigma ,\gamma$, and with fractional-orders $\gamma =1.7,\sigma =0.7$.

**Figure 8:**
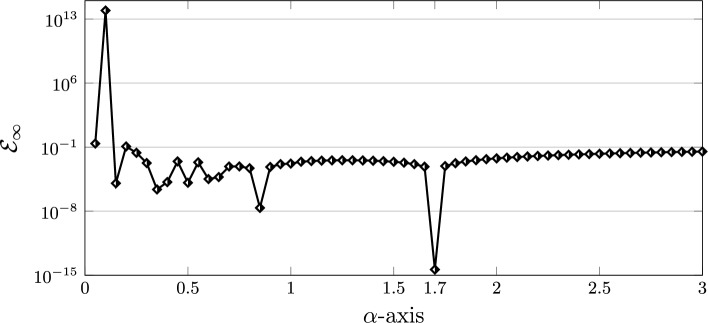
Graphical representation of $\mathcal {E}_{\infty }$ versus $\alpha \in [0.05,3]$ in QLM-FSVLFs approach with $\lambda ,m,Bi=1$, $A=2$, $K=10$, and with fractional-orders $\gamma =1.7,\sigma =0.7$.

Let us compare the errors obtained by using the three different choices of the (local) parameter $\alpha$ equal to $1,\sigma ,\gamma$. Here, we have $\gamma =1.7$ and $\sigma =0.7$. Motivated by the previous works^23,32^, we also here show that the achieved residual error becomes smallest in magnitude as one selects $\alpha =\gamma$. By taking $K=6$, these results are presented in Fig. 7. To see choosing the $\alpha =\gamma$ leads to the best possible result, we further plot the $\mathcal {E}_{\infty }$ obtained by the QLM-FSVLFs technique versus the parameter $\alpha$ as depicted in Fig. 8. These results are calculated with $K=10$ and $\alpha \in [0.05,3]$ with step size 0.05. Indeed, the approximate solution using $\alpha =\gamma =1.7$ and $\sigma =0.7$ with $K=6$ is as follows

$$\begin{aligned} {h}^{(6)}_{6,\frac{17}{10}}(r)&=1.186078708 - 0.001762202606\,r^{\frac{17}{5}} - 0.06590694179\,r^{\frac{17}{10}} - 0.0000021324688\,r^{\frac{34}{5}}\\&\quad -\, 0.00005880221961\,r^{\frac{51}{10}} - 0.00000009967959942\,r^{\frac{17}{2}}. \end{aligned}$$

A comparison is also done in Table 5 when we have the fractional-orders $\gamma =1.7$ and $\sigma =0.7$. In these experiments, we utilize $K=6$, $m,\lambda ,Bi=1$, and $A=2$ as before. For validation, the outcomes of the previously well-established scheme, namely the PLSM^21^ are shown in this table. One can see that by employing the parameter $\alpha$ to be as the fractional order $\gamma$ leads to more accurate results. Our results using this value of $\alpha$ are considerably more accurate than those reported by PLSM.

**Table 5:** Comparing of numerical results/REFs using QLM-FSVLFs technique for $\gamma =1.7,\sigma =0.7$, $m,\lambda =1$, $A=2$, $K=6$, $\alpha =1,1.7$, and various $r\in [0,1]$.

*r*	QLM-FSVLFs				PLSM^21^	
	$h^{(6)}_{6,1}(r)$	${\mathbb {R}}_{6,1}^{(6)}(r)$	$h^{(6)}_{6,\frac{17}{10}}(r)$	${\mathbb {R}}_{6,\frac{17}{10}}^{(6)}(r)$	$\tilde{u}(r)$	Error
0.0	1.18587014	$0.0000_{-0}$	1.186078708050383	$0.0000_{-0}$	1.18592781	$0.000_{-0}$
0.1	1.18480830	$3.6771_{-3}$	1.184762989665008	$3.5729_{-9}$	1.18482122	$1.438_{-3}$
0.2	1.18196899	$1.9265_{-3}$	1.181798785635447	$4.9691_{-9}$	1.18191175	$3.193_{-4}$
0.3	1.17772081	$5.5686_{-4}$	1.177537074846438	$4.1642_{-9}$	1.17761666	$4.895_{-4}$
0.4	1.17228067	$5.7501_{-6}$	1.172118584802979	$2.2938_{-9}$	1.17216020	$6.580_{-6}$
0.5	1.16576606	$6.1427_{-5}$	1.165624795848758	$6.6587_{-10}$	1.16564798	$2.022_{-4}$
0.6	1.15823600	$1.3109_{-6}$	1.158108073063764	$4.6515_{-11}$	1.15812382	$5.054_{-5}$
0.7	1.14972066	$1.9547_{-5}$	1.149603273305191	$5.0383_{-11}$	1.14960897	$3.427_{-4}$
0.8	1.14023972	$5.4729_{-6}$	1.140133388637926	$3.2844_{-11}$	1.14012396	$1.437_{-4}$
0.9	1.12980937	$3.8068_{-6}$	1.129712674036530	$1.7034_{-11}$	1.12969271	$4.717_{-4}$
1.0	1.11843798	$6.5566_{-6}$	1.118348529287161	$1.1143_{-11}$	1.11832927	$3.310_{-5}$

**Figure 9:**
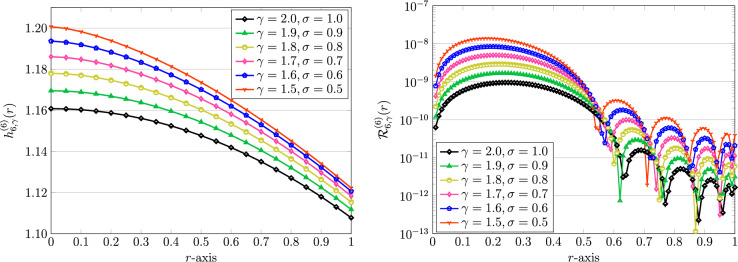
Graphical representation of numerical solutions using QLM-GFSVLFs technique (left) and related REFs (right) with $K=6$, $\alpha =\gamma$, $m,\lambda ,Bi=1$, $A=2$, and with fractional-orders $\gamma ,\sigma$.

Thus, we are motivated to use the parameter $\alpha$ equals to $\gamma$ in the next experiments. With the same problem’s parameters as used in Table 5, various fractional-orders $(\gamma ,\sigma )=(1.5,0.5),(1.6,0.6),\ldots ,(1.9,0.9)$ are utilized in the QLM-GFSVLFs procedure. The graphics are visualized in Fig. 9. It should be noticed that the numerical solution for $(\gamma ,\sigma )=(2,1)$ is further plotted in Fig. 9 to show that the other numerical solutions have tendency to converge to the integer-order cases. In all graphical representations, the value of $\alpha$ is set as $\gamma$. Moreover, the related REFs are plotted alongside the numerical solutions. For different values of $(\gamma ,\sigma )=(1.5,0.5),(1.7,0.7),(1.9,0.9)$ we compute the error norms $\mathcal {E}_{\infty }$ together with order of convergence in Table 6. Clearly, as the fractional-order $(\gamma ,\sigma )$ approaching to (1, 0), the associated $\text{ ord}_K^{\infty }$ will be slightly reduced as expected.

**Table 6:** The results of $\mathcal {E}_{\infty }$ error norms and the corresponding $\text{ ord}_R^{\infty }$ for $m,\lambda ,Bi=1$, $A=2$, $\alpha =\gamma$, and three different $(\gamma ,\sigma )$.

*K*	$(\gamma ,\sigma )=(1.5,0.5)$		$(\gamma ,\sigma )=(1.7,0.7)$		$(\gamma ,\sigma )=(1.9,0.9)$	
	$\mathcal {E}_{\infty }$	$\text{ ord}_K^{\infty }$	$\mathcal {E}_{\infty }$	$\text{ ord}_K^{\infty }$	$\mathcal {E}_{\infty }$	$\text{ ord}_K^{\infty }$
1	$3.6788\times 10^{-1}$	−	$3.6788\times 10^{-1}$	−	$3.6788\times 10^{-1}$	−
2	$7.8809\times 10^{-3}$	5.5447	$7.3924\times 10^{-3}$	5.6371	$6.7762\times 10^{-3}$	5.7626
4	$1.2137\times 10^{-5}$	9.3428	$7.3823\times 10^{-6}$	9.9677	$4.2754\times 10^{-6}$	10.630
8	$1.2088\times 10^{-11}$	19.938	$2.4847\times 10^{-12}$	21.503	$4.6821\times 10^{-13}$	23.122

Table 7 shows some comparisons with the results of the PLSM^21^ in the case of $(\gamma ,\sigma )=(1.8,0.8),(1.9,0.9)$. We utilize $K=6$ and $\alpha =\gamma$ in the computations. In addition to the presented computational results, we report the associated REFs in this table. The outcomes of the recently developed approach PLSM^21^ are brought for comparisons. Note that the errors of the PLSM achieved by using tenth-degree polynomials. The conclusion is that our presented spectral matrix collocation technique with less computational efforts produces more accurate outcomes.

**Table 7:** Comparing of numerical results/REFs using QLM-FSVLFs technique for $(\gamma ,\sigma )=(1.8,0.8),(1.9,0.9)$, $m,\lambda ,Bi=1$, $A=2$, $K=6$, $\alpha =\gamma$, and various $r\in [0,1]$.

*r*	$(\gamma ,\sigma )=(1.8,0.8)$		PLSM^21^		$(\gamma ,\sigma )=(1.9,0.9)$		PLSM^21^	
	$h^{(6)}_{6,\frac{18}{10}}(r)$	${\mathbb {R}}_{6,\frac{18}{10}}^{(6)}(r)$	$\tilde{u}(r)$	Error	$h^{(6)}_{6,\frac{19}{10}}(r)$	${\mathbb {R}}_{6,\frac{19}{10}}^{(6)}(r)$	$\tilde{u}(r)$	Error
0.0	1.17800442	$0.00_{-0}$	1.17790084	$0.000_{-0}$	1.16955055	$0.000_{-0}$	1.16949529	$0.000_{-0}$
0.1	1.17703384	$1.97_{-9}$	1.17703747	$2.175_{-7}$	1.16883733	$1.073_{-9}$	1.16882297	$7.119_{-8}$
0.2	1.17462167	$2.88_{-9}$	1.17466571	$1.703_{-6}$	1.16688696	$1.635_{-9}$	1.16689282	$5.977_{-7}$
0.3	1.17097668	$2.56_{-9}$	1.17101369	$2.094_{-6}$	1.16378969	$1.531_{-9}$	1.16379722	$7.651_{-7}$
0.4	1.16618847	$1.53_{-9}$	1.16621103	$6.919_{-6}$	1.15958555	$9.767_{-10}$	1.15959017	$2.602_{-6}$
0.5	1.16030974	$5.15_{-10}$	1.16032450	$3.970_{-6}$	1.15429709	$3.718_{-10}$	1.15430000	$1.527_{-6}$
0.6	1.15337399	$6.89_{-12}$	1.15338557	$6.381_{-6}$	1.14793690	$2.861_{-11}$	1.14793934	$2.498_{-6}$
0.7	1.14540258	$3.77_{-11}$	1.14540992	$1.768_{-6}$	1.14051068	$2.495_{-11}$	1.14051221	$7.030_{-7}$
0.8	1.13640822	$1.77_{-11}$	1.13640877	$1.292_{-6}$	1.13201881	$8.855_{-12}$	1.13201843	$5.204_{-7}$
0.9	1.12639692	$8.52_{-12}$	1.12639214	$1.402_{-7}$	1.12245723	$4.044_{-12}$	1.12245516	$5.716_{-8}$
1.0	1.11536923	$5.92_{-12}$	1.11536400	$5.497_{-8}$	1.11181790	$3.125_{-12}$	1.11181564	$2.264_{-8}$

Ultimately, we fix the fractional-order parameters given by

$$\begin{aligned} \gamma =1.75,\quad \sigma =0.75. \end{aligned}$$

**Figure 10:**
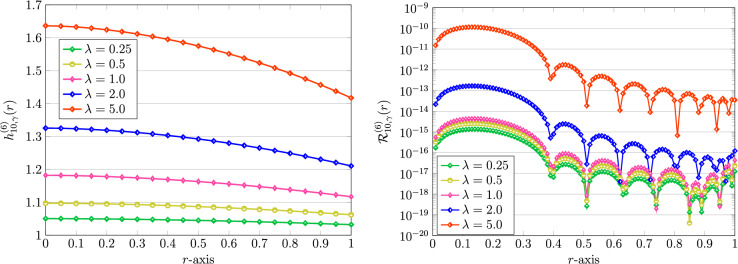
Graphical representation of numerical solutions using QLM-FSVLFs technique (left) and related REFs (right) with $(\gamma ,\sigma )=(1.75,0.75)$, $K=10$, $\alpha =\gamma$, $m,Bi=1$, $A=2$ and various $\lambda$.

**Figure 11:**
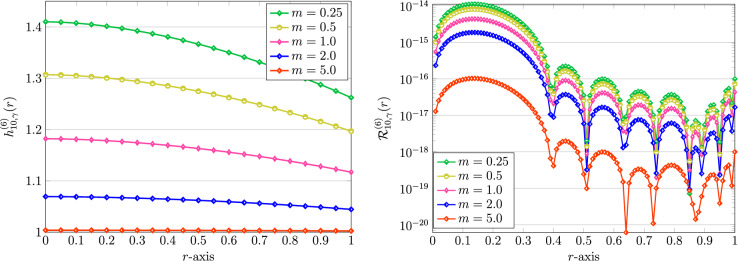
Graphical representation of approximate solutions by using QLM-FSVLFs technique (left) and associated REFs (right) with $(\gamma ,\sigma )=(1.75,0.75)$, $K=10$, $\alpha =\gamma$, $\lambda ,Bi=1$, $A=2$ and various *m*.

Then, we consider the impacts of diverse parameters $m,\lambda ,Bi$ on the approximate solutions of the model equation (1.4) through the QLM-FSVLFs method. To this end, we set $K=10$ and use $\alpha =\gamma$, which is 1.75. Also, we take $A=2$ in all subsequent experiments. First, we vary $\lambda =0.25,0.5,1,2,5$ and fix other parameters as $m,Bi=1$. The results of approximations are plotted on the left part of Fig. 10. Furthermore, on the right part, the results of REEFs are presented. As in the integer-order cases, by increasing the nonlinearity parameter $\lambda$, the achieved errors get increased. In the next experiments, we will consider the effect of the nonlinearity parameter *m*. In this respect, we vary $m=0.25,0.5,1,2,5$. The outcomes of numerical solutions along with the associated REFs are shown in Fig. 11. Obviously, the magnitude of the achieved errors will be decreased if the factor of nonlinearity *m* is increased in the model of human head.

In the final stage, let us investigate the effects of using the boundary condition parameter *Bi* on the calculations of QLM-GFSVLFs. The $L_{\infty }$ error norms (4.19) together with the corresponding numerical order of convergences calculated via (4.20) are further shown in Table 8 for the values of $m=0.25,0.5,1,2,5$. It can be evidently seen that the developed QLM-FSVLFs technique produces high-order accurate outcomes when applied to the model (1.4).

**Table 8:** The results of $\mathcal {E}_{\infty }$ error norms and the corresponding $\text{ ord}_R^{\infty }$ for $m,\lambda =1,$ $A=2$, $(\gamma ,\sigma =(1.75,0.75)$, $\alpha =\gamma$, and different *Bi*, *K*.

*K*	$Bi=0.5$		$Bi=1$		$Bi=2$		$Bi=5$	
	$\mathcal {E}_{\infty }$	$\text{ ord}_R^{\infty }$	$\mathcal {E}_{\infty }$	$\text{ ord}_R^{\infty }$	$\mathcal {E}_{\infty }$	$\text{ ord}_R^{\infty }$	$\mathcal {E}_{\infty }$	$\text{ ord}_R^{\infty }$
1	$3.6788_{-1}$	−	$3.6788_{-1}$	−	$3.6788_{-1}$	−	$3.6788_{-1}$	−
2	$5.9895_{-3}$	5.9407	$7.2480_{-3}$	5.6655	$8.0910_{-3}$	5.5068	$8.6963_{-3}$	5.4027
4	$4.4530_{-6}$	10.393	$6.4672_{-6}$	10.130	$8.0143_{-6}$	9.9795	$9.2211_{-6}$	9.8812
8	$7.8675_{-13}$	22.432	$1.6509_{-12}$	21.901	$2.5276_{-12}$	21.596	$3.3394_{-12}$	21.397

## Conclusions

A numerical spectral collocation procedure relied on the (novel) fractional-order shifted Vieta–Lucas functions (FSVLFs) designed to get the polynomial solutions to a class of nonlinear fractional-order two-point boundary value problems with singularity arising in modeling of heat conduction of the human head. Both fractional derivative operators are interpreted in the sense of Liouville–Caputo’s derivative. To conquer the inherit nonlinearity of the model problem, the quasilinearization method (QLM) is utilized to reach at a family of linearized subequations. Hence, we solved this family of equations through using the spectral matrix collocation scheme relied on the FSVLFs. Theoretically, we proved that the related series solution of FSVLFs is convergent in the weighted $L_2$ norm. In particular, we showed that the series of FSVLFs is uniformly convergent of order $\mathcal {O}(K^{-3})$, where *K* is the number of FSVLFs used in the approximation. Simulation results are provided to verify our theoretical findings and illustrate the effectiveness of the presented QLM-FSVLFs scheme. Numerical results are well tabulated through tables against the various model parameters $m,\lambda ,Bi,A$ as well as fractional orders $\gamma$ and $\sigma$. We have compared our outcomes and plots with those reported by the PLSM^21^ and there is a good level of agreement in our results. The reported rate of convergence shows that the QLM-FSVLFs method converges exponentially as the number of bases increases. The proposed QLM-FSVLFs technique with high-order accuracy can be easily generalized to tackle various multi-order multi-dimensional fractional model problems with applications in various disciplines of science and engineering.

## Data Availability

Data sharing not applicable to this article as no datasets were generated or analyzed during the current study.
